# Complete Genome Sequence of Pseudomonas aeruginosa Reference Strain PAK

**DOI:** 10.1128/MRA.00865-19

**Published:** 2019-10-10

**Authors:** Amy K. Cain, Laura M. Nolan, Geraldine J. Sullivan, Cynthia B. Whitchurch, Alain Filloux, Julian Parkhill

**Affiliations:** a Wellcome Sanger Institute, Wellcome Trust Genome Campus, Hinxton, Cambridge, United Kingdom; b Department of Molecular Sciences, Macquarie University, North Ryde, Australia; c Imperial College London, MRC Centre for Molecular Bacteriology and Infection, South Kensington, London, United Kingdom; d The ithree institute, Faculty of Science, University of Technology Sydney, Ultimo, Australia; e Department of Veterinary Medicine, University of Cambridge, Cambridge, United Kingdom; University of Arizona

## Abstract

We report the complete genome of Pseudomonas aeruginosa strain PAK, a strain which has been instrumental in the study of a range of P. aeruginosa virulence and pathogenesis factors and has been used for over 50 years as a laboratory reference strain.

## ANNOUNCEMENT

Pseudomonas aeruginosa is one of the deadly and highly drug-resistant, hospital-associated ESKAPE pathogens (Enterococcus faecium, Staphylococcus aureus, Klebsiella pneumoniae, Acinetobacter baumannii, Pseudomonas aeruginosa, and Enterobacter species), and it has been flagged by the WHO as a species of “greatest public health concern.” P. aeruginosa strain K (PAK) is one of the most well-studied P. aeruginosa laboratory strains, first described for its Pf1 phage sensitivity in 1966 (1) and its hyperpiliation in 1972 (2), and it is still in use today. This virulent exemplar strain highly expresses pili and flagella and contains glycosylation and pathogenicity islands (3, 4). Although over 500 publications involving the PAK strain exist to date (PubMed database), a long-read, closed, and high-quality genome annotation sequence is lacking.

Whole-genomic PAK DNA was sourced from the Filloux laboratory collection at Imperial College London, which originally was kindly gifted by Stephen Lory at Harvard Medical School. The strain was revived from storage in glycerol at −80˚C by streaking the bacteria onto an LB agar plate and growing it overnight. A single colony was then selected for overnight growth at 37˚C in LB broth, with continuous shaking. The DNA was prepared using a phenol-chloroform method (5), and 2 μg was sequenced using the long-read Pacific Biosciences RS II sequencing platform (PacBio, USA). Genomic DNA (gDNA) was needle sheared to 26,522 bp (4 complete passes using a 26-gauge 2-in. blunt-ended needle), and the library was sequenced using the manufacturer’s protocol with the P4-C2 chemistry kit on 5 single-molecule real-time (SMRT) cells, producing 126,553 total reads and an average read length of 3,494 bp. *De novo* assembly of these reads was performed using the Hierarchical Genome Assembly Process 3 (HGAP3), on the SMRT Analysis pipeline 2.2.0, into a single contig with 67.2× coverage, which was linearized at *dnaA* using Circlator (6).

**FIG 1:**
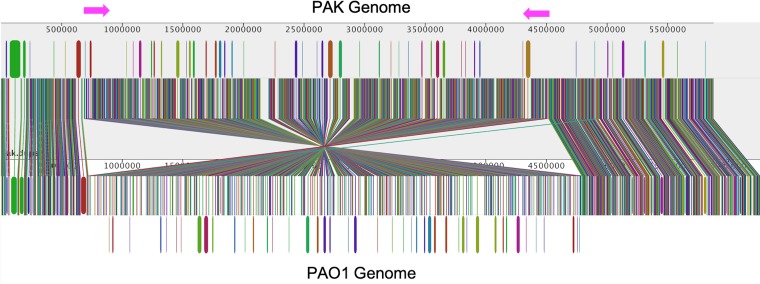
Comparison of the Pseudomonas aeruginosa strain PAK genome and reference strain PAO1 genome. Sequences were linearized at position 1 of PAK and visualized using Mauve (13). Orthologous regions between the genomes are indicated by the locally colinear blocks matched by vertical lines. Inverted magenta arrows mark the potential rRNA inversion point (arrows not to scale).

The PAK chromosome consists of 6,395,872 bp and contains 5,757 coding DNA sequences (CDSs) and 111 RNAs, with an average GC content of 66.44%. Default parameters were used for all software unless otherwise specified. Using the *Pseudomonas* multilocus sequence type (MLST) database (7), PAK was categorized as sequence type 693 (ST693). *De novo* annotation was performed using PROKKA 1.12 (8), followed by a secondary annotation transfer from reference strain PAO1 with RATT (9) using a cutoff of 90% similarity. Comparisons of PAK with reference strain PAO1 revealed 44,127 unique single nucleotide polymorphisms (SNPs), determined using SNP-calling methods as previously described (10), and identified 5,537 orthologous genes, as defined by BLASTN (11). We identified a large inversion of 4,185,263 bp between the strains using ACT (12), and visualized it using Mauve (13) (Fig. 1). Two sets of complementary rRNA genes of 5,752 bp with 99.63% sequence identity flank the inversion site.

Further manual annotation was performed. Five acquired resistance genes were identified using ResFinder 2.0 (14), all of which are also in PAO1, as follows: *aph(3′)-IIb* (aminoglycoside), *ampC*, *bla*_OXA-50_ (beta-lactamase), *catB7* (chloramphenicol), and *fosA* (fosfomycin). PHASTER (15) was used to identify the bacteriophage Pf1. Interestingly, Pf1 has long been known to be quite specific for PAK, yet Pf1 does not always integrate into the PAK chromosome (16). IslandViewer 4 (17) found 8 further genomic islands. The type IV pilus genes *pilA* to *pilD*, *pilF*, *pilG* to *pilK*, *chpA* to *chpC*, *fimL*, *fimS*, *algR*, *fimT, fimU, pilV* to *pilX, pilY1, pilY2, pilE*, and *pilM* to *pilQ* and the 3 type VI secretion system (T6SS) clusters, H1, H2, and H3 were identified. The H1 cluster spanned from *tagQ1* to PA0101, the H2 cluster from *tssA2* to *stk1*, and the H3 cluster from *sfa3* to PA2375.

This high-quality genome sequence will advance future understanding of this pathogen and help integrate almost 50 years of research on PAK into a modern genomic context.

### Data availability.

This whole-genome sequence has been deposited in GenBank under accession number LR657304, with the raw PacBio reads accessible under European Nucleotide Archive (ENA) sample accession number ERS484051.
